# Meta‐analysis of surgical treatment for postinfarction left ventricular free‐wall rupture

**DOI:** 10.1111/jocs.15701

**Published:** 2021-06-01

**Authors:** Matteo Matteucci, Francesco Formica, Mariusz Kowalewski, Giulio Massimi, Daniele Ronco, Cesare Beghi, Roberto Lorusso

**Affiliations:** ^1^ Department of Cardiothoracic Surgery, Heart and Vascular Centre Maastricht University Medical Centre Maastricht The Netherlands; ^2^ Department of Surgical and Morphological Sciences, Circolo Hospital University of Insubria Varese Italy; ^3^ Unit of Cardiac Surgery, Department of Medicine and Surgery University of Parma, University Hospital of Parma Parma Italy; ^4^ Department of Cardiac Surgery Central Clinical Hospital of the Ministry of Interior in Warsaw Warsaw Poland; ^5^ Department of Cardiac Surgery Niguarda Hospital Milan Italy; ^6^ Unit of Cardiac Surgery, Department of Cardio‐Thoracic and Vascular Niguarda Hospital Milan Italy

**Keywords:** acute myocardial infarction, surgical repair, ventricular rupture

## Abstract

**Background:**

Left ventricular free‐wall rupture (LVFWR) is one of the most lethal complications after acute myocardial infarction (AMI). The optimal therapeutic strategy is controversial. The current meta‐analysis sought to examine the outcome of patients surgically treated for post‐AMI LVFWR.

**Methods:**

A comprehensive literature review was performed to identify articles reporting outcomes of subjects who underwent LVFWR surgical repair. The primary endpoint was operative mortality. A meta‐analysis was performed to assess the associations of predefined variables of interest and clinical prognosis.

**Results:**

Of the 3132 retrieved articles, 11 nonrandomized studies, enrolling a total of 363 patients, fulfilled the inclusion criteria and were included in this analysis. The mean age of patients was 68 years. The operative mortality rate was 32% (*n* = 115). Meta‐analysis revealed reduced operative risk in patients with oozing type rupture, as compared to blowout type (risk ratios [RR]: 0.47; 95% confidence interval [CI]: 0.33–0.67; *p* < .0001); RR was also significantly reduced in subjects in whom LVFWR was treated with sutureless technique, as compared to those undergoing sutured repair (RR: 0.59; 95% CI: 0.41–0.83; *p* = .002). Increased risk of operative mortality was demonstrated in patients who required postoperative extracorporeal membrane oxygenation (ECMO) support (RR: 2.39; 95% CI: 1.59–3.60; *p* < .0001).

**Conclusions:**

Surgical treatment of postinfarction LVFWR has a high operative mortality rate. Blowout rupture, sutured repair and postoperative ECMO support are factors associated with increased risk of operative mortality.

## 1 INTRODUCTION

Left ventricular free‐wall rupture (LVFWR) is a known complication of acute myocardial infarction (AMI). Advances in reperfusion strategies, such as percutaneous coronary intervention, have resulted in significant decrease in the rates of postinfarction mechanical complications of AMI.^1^, ^2^ In the prethrombolytic era, LVFWR was thought to complicate 2%–4% of AMI presentations.^3^ Contemporary registries, however, show it to be increasingly uncommon, complicating between 0.01% and 0.5% of subjects presenting with AMI.^1^, ^2^ Unfortunately though, despite a declining incidence, postinfarction LVFWR still portends an ominous prognosis, with mortality rates between 39% and 92% in current series.^4^, ^5^, ^6^ Surgery is the definitive therapy for LVFWR, and aims to close the myocardial tear and prevent a recurrent rupture or pseudoaneurysms formation.^7^ However, due to the rarity of this post‐AMI event, in the literature only little information on the surgical treatment for post‐AMI LVFWR are available. The purpose of this meta‐analysis is to synthesize the current evidence regarding LVFWR repair, and to determine risk factors affecting the early outcome of these patients.

## 2 METHODS

### 2.1 Search strategy

The current meta‐analysis was performed in accordance with the PRISMA (Preferred Reporting Items for Systematic Reviews and Meta‐Analyses statement).^8^ The study protocol was registered and published online in PROSPERO (The International Prospective Register of Systematic Reviews) (CRD42021225611). Online databases (PubMed, Embase, and the Cochrane Central Register of Controlled Trials) were screened from January 1, 1990 to November 30, 2020 for relevant reports. In addition, reference lists were carefully analysed and cross‐checked for articles that escaped the searches in electronic databases. Keywords pertinent to the exposure of interest were used in relevant combinations: “ventricular free‐wall rupture,” “cardiac rupture,” “heart rupture,” “myocardial rupture,” “acute myocardial infarction,” “postinfarction mechanical complication.” The language was limited to English.

### 2.2 Study selection criteria

Retrospective observational cohort studies of adult (>18 years old) undergoing cardiac surgery for post‐AMI LVFWR were eligible for inclusion in the analysis. Exclusion criteria were as follows^1^: animal studies;^2^ LVFWR not AMI‐related (e.g., posttraumatic);^3^ reports not reporting operative mortality^4^ studies including less than 10 surgical patients. Systematic reviews were not considered. Two investigators (M.M. and F.F.) independently screened titles and abstracts. After excluding nonrelevant reports, full texts of potentially relevant studies were then screened for inclusion in the final analysis. A standardized form was used to extract data from included studies for assessment of study quality and evidence synthesis. Any divergences were resolved by consensus.

Risk of bias at the individual study level was appraised with ROBINS‐I (Risk of Bias in Not Randomized Studies of Interventions), a tool used for assessment of the bias (the selection of the participants; the ascertainment of either the exposure or outcome of interest; etc.) in cohort studies included in a meta‐analysis.^9^ Overall quality was independently determined by two reviewers (F.F. and G.M.); discrepancies were resolved by discussion and adjudication by a third reviewer (M.M.).

### 2.3 Statistical analysis

The outcome measure was operative mortality, defined as any death, regardless of cause, occurring within 30 days after surgery (in or out of hospital) or after 30 days but during the index hospitalization subsequent to the surgery. The meta‐analysis was performed using Review Manager (RevMan) 5.3 software, from the Cochrane Collaboration (The Nordic Cochrane Centre, Copenhagen, Denmark). Inverse variance random‐effects model analysis using the proportions of patients who experienced the outcome of interest (i.e., operative mortality) was performed. Pooled effect estimates were expressed as risk ratios (RR) with 95% confidence interval (CI), and the probability for overall effect was deemed significant if *p* < .05. Heterogeneity among the included reports was assessed using both the Cochran's *Q* test and the *I* ^2^. Significant heterogeneity was considered for a *p* < .1 at the *Q* statistic. We defined heterogeneity as follows: *I* ^2^ = 0%–49%, low heterogeneity; *I* ^2^ = 50%–74%, moderate heterogeneity; and *I* ^2^ > 75%, severe heterogeneity. Sensitivity analysis was carried out by successively excluding the low‐quality studies to assess the stability of the outcome. Publication bias was evaluated by the visual assessment of funnel plots; asymmetric funnel plot indicated possible publication bias.

## 3 RESULTS

### 3.1 Search results and bias

The study selection process and reasons for exclusion are described in Figure S1. After removal of articles not pertinent to the design of the current study, 11 reports^10^, ^11^, ^12^, ^13^, ^14^, ^15^, ^16^, ^17^, ^18^, ^19^, ^20^ with suitable data were included in the final meta‐analysis. All selected papers were published after 2001. The number of included patients for each trial ranged from 14 to 140, with a total of 363 subjects. Table 1 summarizes the main characteristics of the included studies. Potential sources of the studies' bias were analyzed with the use of components recommended by the ROBINS‐I tool, and the results are enclosed as Table S1. Overall, the studies reported moderate or serious risk of bias. However, the analysis of the funnel plots suggested that the risk of publication bias was low (Figures S2 and S3).

**Table 1:** Studies and patients' baseline characteristics

Author (ref)	Total patients	Age (year)	Pre‐op IABP	Ant/apic rupture	Blowout rupture	Sutured repair	Conc CABG	On‐pump repair	Post‐op IABP	Post‐op ECMO	Operative mortality
Kacer (2020)^10^	19	64	1	4	n.a.	14	7	17	4	1	5
Matteucci (2020)^11^	140	69	51	47	61	86	34	84	67	11	51
Okamura (2019) ^12^	35	72	13	16	2	7	3	0	n.a.	n.a.	6
Formica (2017)^13^	35	68	14	6	16	19	15	27	10	11	12
Zoffoli (2012)^14^	25	65	0	n.a.	n.a.	4	2	n.a.	n.a.	3	3
Haddadin (2009)^15^	19	72	8	7	11	16	4	13	6	n.a.	5
Okada (2005)^16^	14	74	8	n.a.	8	6	4	7	n.a.	n.a.	9
Flajsig (2002)^17^	24	62	5	14	n.a.	19	n.a.	11	n.a.	n.a.	8
Mantovani (2002)^18^	17	68	7	3	n.a.	16	11	17	n.a.	0	3
Iemura (2001)^19^	17	65	4	12	3	10	8	12	15	n.a.	2
McMullan (2001)^20^	18	64	0	n.a.	14	16	4	18	6	n.a.	11
Total	363	68	111	109	115	213	92	206	108	26	115
(% or ± *SD*)	(100)	(±4)	(31)	(36)	(41)	(59)	(27)	(61)	(44)	(11)	^32^

*Note*: Data are shown as mean or number, as appropriate.

Abbreviations: ant/apic, antero/apical; conc CABG, concomitant coronary artery bypass grafting; ECMO, extracorporeal membrane oxygenation; IABP, intraaortic balloon pump; n.a., not available; pre‐op, preoperative; Ref, reference; y, years.

### 3.2 Participant characteristics and outcome

The mean age of patients was 68 ± 4 years. Nearly one‐third of patients were supported with intraaortic balloon pump (IABP) preoperatively (Table 1). The oozing type was the most common rupture (59%) encountered at the time of surgery, whereas sutured repair was the technique most frequently used (59%) to treat LVFWR. In 36% of cases, the rupture involved the antero‐apical wall, in the remaining patients the locations were in the lateral or posterior wall. Most commonly post‐AMI LVFWR was treated (66%) utilizing cardiopulmonary bypass (CPB); only 27% of subjects had concomitant coronary artery bypass grafting (CABG) at the time of LVFWR repair. Postoperative IABP support was necessary in almost 45% of the patients, whereas only 11% of individuals required extracorporeal membrane oxygenation (ECMO) after surgery. Overall, the total number of early deaths was 115, representing an operative mortality rate of 32%.

### 3.3 Meta‐analysis

**Figure 1:**
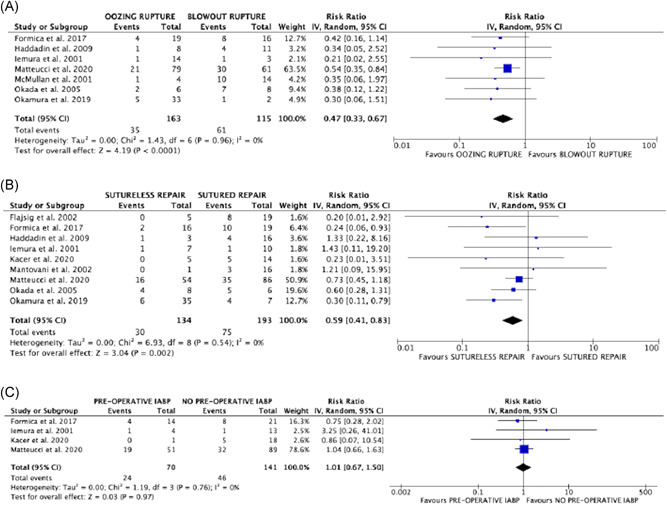
Forrest plots of comparison (from above to below): (A) oozing rupture versus blowout rupture; (B) sutureless repair versus sutured repair; (C) preoperative IABP support versus no IABP support; outcome of interest: operative mortality. IABP, intraaortic balloon pump

**Figure 2:**
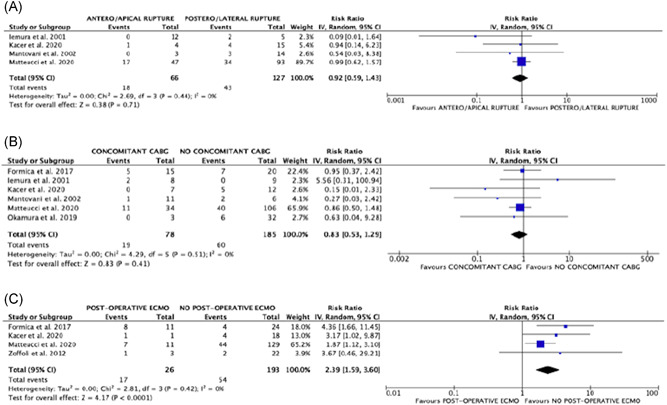
Forrest plots of comparison (from above to below): (A) antero‐apical rupture versus postero‐lateral rupture; (B) concomitant CABG versus no CABG; (C) postoperative ECMO support versus no ECMO support; outcome of interest: operative mortality. CABG, coronary artery bypass grafting; ECMO, extracorporeal membrane oxygenation

**Figure 3:**
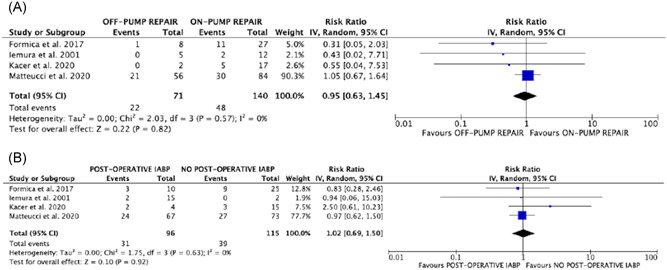
Forrest plots of comparison (from above to below): (A) on‐pump repair versus off‐pump repair; postoperative IABP support versus no IABP support; outcome of interest: operative mortality. IABP, intraaortic balloon pump

Risks of operative mortality were significantly reduced in subjects with oozing type rupture, as compared to blowout type (RR: 0.47; 95% CI: 0.33–0.67; *p* < .0001; *I* ^2^ = 0% (Figure 1), with early death rates of 21.5% (35/163) and 53% (61/115), respectively. Risks were also significantly reduced in patients in whom LVFWR was treated with sutureless technique, as compare to those underwent sutured repair (RR: 0.59; 95% CI: 0.41–0.83; *p* = .002; *I* ^2^ = 0% (Figure 1), corresponding rates of death were 22.4% (30/134) and 38.9% (75/193). There was no significant difference in the risks of early mortality between patients with or without preoperative IABP support (RR: 1.01; 95% CI: 0.67–1.50; *p* = .97) and between subjects with antero‐apical or postero‐lateral wall rupture (RR: 0.92; 95% CI: 0.59–1.43; *p* = .71), with no heterogeneity among studies (Figures 1 and 2). A no‐significant trend towards reduced risks of operative mortality was observed when the repair was performed with concomitant CABG (RR: 0.83; 95% CI: 0.53–1.29; *p* = .41; *I* ^2^ = 0% (Figure 2), whereas increased risks were seen in patients with postoperative ECMO support (RR: 2.39; 95% CI: 1.59–3.60; *p* < .0001; *I* ^2^ = 0% (Figure 2). No significant differences in the risks of operative death were found in patients who required CPB for LVFW repair and in subjects who needed postoperative IABP, as compared to their counterparts (RR: 0.95; 95% CI: 0.63–1.45; *p* = .82; *I* ^2^ = 0% and RR: 1.02; 95% CI: 0.69–1.50; *p* = .92; *I* ^2^ = 0%, respectively (Figure 3).

### 3.4 Sensitivity analysis

Analysis performed by deleting studies at highest risk of bias, and one study in turn did not reveal any change in direction nor magnitude of the treatment effect.

## 4 DISCUSSION

LVFWR following AMI is still a challenging complication with high associated mortality; when treated, provided prompt diagnosis and immediate surgery, expected mortality is only modestly improved. Indeed, despite the innovation in reperfusion strategies, technologies and surgical techniques, the mortality and morbidity remain as high as more than 40%.^4^, ^5^, ^6^

Subjects with LVFWR represent a high‐risk population among those who have suffered from AMI.

Hemodynamic instability that often leads to cardiogenic shock, cardiac tamponade which is often complicated by cardiocirculatory collapse, the friable tissue surrounding the infarct area which can rapidly evolve to a large and nonsurgically reparable rupture are all factors that influence dramatically the operative mortality. The Global Registry of Acute Coronary Events^4^ and more recently other authors^6^ reported mortality rates ranging from 80% to 92% in medically managed LVFWR patients. Therefore, surgical repair is considered the standard of care for this condition, although surgery remains a challenging operation often correlated with a complicated postoperative course.

Given very low rates of presentation and lack of both randomized studies due to ethical reasons and large multicenter trials, we aimed to overcome this gap giving an overview of published data and results following surgery after LVFWR repair. This is the first review, to our knowledge, to address factors associated with mortality following LVFWR.

Surgical repair of LVFWR has evolved over time mainly with the advent of tissue adhesive biological glues which allow to secure different typology of patches, both biological (autologous or heterologous pericardium) or synthetic (Dacron, Teflon) to cover the rupture myocardial area. More recently, collagen sponge patches such as TachoSil® or TachoComb® were described as a valid alternative to patch covering.^7^, ^12^, ^21^, ^22^, ^23^ These strategies are usually preferred in selected patients, mainly in such conditions were oozing rupture is observed during surgery. Regarding this issue, another study by our group found a significant protective effect of the named “patch and glue” sutureless technique over the sutured technique, previously described.^7^ We found that the sutureless technique allows a reduction of about 40% of the in‐hospital mortality compared to the suture technique, which was reported in 213 patients (59%). Moreover, we have observed a protective effect of the oozing rupture compared to the blowout one, the former reducing the in‐hospital mortality of about 50% compared to the blowout rupture presentation. Since not all LVFWR can be dealt with in a sutureless fashion, these findings must be viewed with caution. However, we can speculate that the use of sutureless technique is widely preferred and applied in patients presenting with oozing rupture due to the limited myocardial damage.^24^ In this anatomical feature, which is often associate with hemodynamic stable condition during surgery, operation would be easily performed without CPB to reduce its adverse effects. However, when we focused on the correlation between the use of CPB and in‐hospital mortality, we have no found any difference compared to patients who underwent surgery without CPB.

A topic worthy of further discussion concerns the preoperative or intraoperative IABP insertion which is advocated by some authors to obtain a hemodynamic stabilization before surgery^25^ and even in the absence of haemodynamic instability.^26^ The IABP increases the coronary blood flow and reduces the intracavitary pressure of the left ventricle and therefore, in such critical conditions, can reduce both the infarct extension and the incidence of transition from oozing to blowout rupture^27^ and to prevent re‐rupture following surgery^28^ as well. Our analysis revealed that IAPB was preoperatively inserted in 111 patients (31%), while the use of postoperative IABP was reported in 6 among 11 studies included in this meta‐analysis for a total of 108/248 patients (44%). We failed to demonstrate any protective effect of preoperative or postoperative insertion of IABP in terms of operative mortality.

The implantation of veno‐arterial (VA) ECMO is still controversial due to the lack of large data and the poor and discouraging results published so far. Even an outcome analysis of the Extracorporeal Life Support Organization Registry failed to report consistent results concerning the use of VA‐ECMO in LVFWR.^29^ We can speculate that the use of VA‐ECMO in such complex clinical scenario is always considered as the last resort for the patient and it is applied preoperatively often during cardiopulmonary resuscitation maneuvers and perioperatively because of CPB weaning failure. Formica et al.^13^ reported a relatively high incidence of brain death among patients who received VA‐ECMO for cardiac arrest at presentation. Moreover, multivariable analysis identified only cardiac arrest at presentation as an independent predictor of in‐hospital mortality. In these patients, it was not possible to verify the proper utility of the VA‐ECMO support because cardiac death happened before starting a weaning protocol. On the other hand, 17.4% of patients who received VA‐ECMO for cardiac arrest at presentation survived to surgery.^21^ This study found a clear negative effect of the postoperative VA‐ECMO insertion with an increasing of 2.4‐fold the odds of operative mortality. However, given the retrospective nature of the study, one can hypothesize that most of patients requiring postoperative VA‐ECMO are part of the worst subgroup in terms of extension of myocardial infarction, myocardial tissue damage and irreversible brain damage and in such clinical scenario VA‐ECMO institution maybe sometimes considered as compassionate use. Although the indications for VA‐ECMO support are widely recognized, as well as the weaning and management strategies, in this meta‐analysis it was not possible to identify the appropriate indications for the use of VA‐ECMO in the postoperative period for two main reasons: first, few patients (26/363, 11%) received postoperative VA‐ECMO support and of these it was not possible to identify exactly the causes determining the need for the mechanical support besides the CPB weaning failure; second, none of the four studies (10, 11, 13, 14) that reported the postoperative VA‐ECMO implant have focused on the indications and management strategies such as weaning due to myocardial recovery, bridging to the heart transplant or to long‐term left ventricular assist device. Further studies are needed to address this issue thoroughly.

## 5 LIMITATIONS

This meta‐analysis is affected by the common limitations regarding meta‐analysis of observational studies. In particular, the quality of the studies included for the analysis represents the main limitation. There were no prospective or randomized studies included and only one study was a retrospective multicenter trial. Furthermore, they are limited by a relatively small number of patients. Long‐term follow‐up analysis was not considered due to limited amount of data. Another limitation concerns the lack of some preoperative variables, such as the left ventricular ejection fraction at presentation and the pericardial drainage in patients presenting or developing massive pericardial effusion or tamponade which might have impacted the postoperative outcome.

## 6 CONCLUSIONS

LVFWR following AMI is a rare complication affected by a high mortality despite prompt diagnosis and surgical treatment. The standard of care is emergency surgical repair. Sutureless repair is the best option treatment when possible and oozing rupture confers a higher probability of early survival compared to blowout rupture. However, the more recent literature provided limited and poor data, and therefore, further studies are required to provide additional data and evidence in terms of early and long‐term results.

## CONFLICT OF INTERESTS

Roberto Lorusso consultant for Medtronic and LivaNova, and member of the Advisory Board of Eurosets and PulseCath. Other authors have no conflict of interests.

## Supporting information

Supporting information.
