# Supplementary material for: Meta‐analysis of surgical treatment for postinfarction left ventricular free‐wall rupture
Source: J Card Surg. 2021 Jun 1;36(9):3326–33. doi: 10.1111/jocs.15701 (PMC8453579; doi:10.1111/jocs.15701)
Supplement: Supplementary file 1 — Supporting information. [file JOCS-36-3326-s001.docx]

**Supplemental Material**

**Meta-Analysis of Surgical Treatment for Post-Infarction Left Ventricular Free-Wall Rupture**

Matteo Matteucci, MD, Francesco Formica, MD, Mariusz Kowalewski, MD, Giulio Massimi, MD, Daniele Ronco, MD, Cesare Beghi, MD, Roberto Lorusso, MD, PhD.

**Supplemental Figure**

Figure 1. The PRISMA flow diagram……………………………………..…….....……………page 2

Figure 2. Publication bias (I) ........................................................................................................page 3

Figure 3. Publication bias (II) ......................................................................................................page 4

**Supplemental Table**

Table 1. Risk of bias summary....................................................................................................page 5

**Figure 1. The PRISMA flow diagram**

The PRISMA flow diagram describing the study selection process.

**Figure 2. Publication bias (I)**

**Figure 3. Publication bias (II)**

**Table 1. Risk of bias summary**

| Study | Bias due to  confounding | Bias in selection of participants | Bias in measurement of interventions | Bias due to missing data | Bias in measurement of outcomes | Bias in selection of reported results | Overall bias |
| --- | --- | --- | --- | --- | --- | --- | --- |
| *Kacer et al.* | Serious | Low | Low | Moderate | Moderate | Moderate | Moderate |
| *Matteucci et al.* | Serious | Low | Low | Low | Moderate | Moderate | Moderate |
| *Okamura et al.* | Serious | Low | Moderate | Moderate | Moderate | Moderate | Moderate |
| *Formica et al.* | Serious | Low | Moderate | Low | Moderate | Moderate | Moderate |
| *Zoffoli et al.* | Serious | Moderate | Moderate | Moderate | Serious | Moderate | Moderate |
| *Haddadin et al.* | Serious | Low | Low | Moderate | Moderate | Moderate | Moderate |
| *Okada et al.* | Serious | Low | Moderate | Moderate | Serious | Serious | Serious |
| *Flajsig et al.* | Serious | Low | Moderate | Moderate | Serious | Serious | Serious |
| *Mantovani et al.* | Serious | Moderate | Moderate | Serious | Moderate | Moderate | Moderate |
| *Iemura et al.* | Serious | Moderate | Serious | Moderate | Moderate | Moderate | Moderate |
| *McMullan et al.* | Serious | Moderate | Moderate | Serious | Moderate | Serious | Serious |

Review authors' judgements about each risk of bias item for each included study.
